# Brain glycogen build-up measured by magnetic resonance spectroscopy in classic infantile Pompe disease

**DOI:** 10.1093/braincomms/fcae303

**Published:** 2024-09-12

**Authors:** Chloé Najac, Nadine A M E van der Beek, Vincent O Boer, Pieter A van Doorn, Ans T van der Ploeg, Itamar Ronen, Hermien E Kan, Johanna M P van den Hout

**Affiliations:** C.J. Gorter MRI Center, Department of Radiology, Leiden University Medical Center, 2333 ZA Leiden, The Netherlands; Center for Lysosomal and Metabolic Diseases, Department of Neurology, Erasmus MC University Medical Center, 3000 CA Rotterdam, The Netherlands; Danish Research Center for Magnetic Resonance, Centre for Functional and Diagnostic Imaging and Research, Copenhagen University Hospital Amager and Hvidovre, DK2650 Copenhagen, Denmark; Center for Lysosomal and Metabolic Diseases, Department of Neurology, Erasmus MC University Medical Center, 3000 CA Rotterdam, The Netherlands; Center for Lysosomal and Metabolic Diseases, Department of Pediatrics, Erasmus MC University Medical Center, 3000 CA Rotterdam, The Netherlands; Clinical Imaging Sciences Centre, Brighton and Sussex Medical School, Brighton, East Sussex BN1 9RR, UK; C.J. Gorter MRI Center, Department of Radiology, Leiden University Medical Center, 2333 ZA Leiden, The Netherlands; Duchenne Center Netherlands, 2333 ZA Leiden, The Netherlands; Center for Lysosomal and Metabolic Diseases, Department of Pediatrics, Erasmus MC University Medical Center, 3000 CA Rotterdam, The Netherlands

**Keywords:** brain, glycogen, magnetic resonance spectroscopy, Pompe disease

## Abstract

Classic infantile Pompe disease is caused by abnormal lysosomal glycogen accumulation in multiple tissues, including the brain due to a deficit in acid α-glucosidase. Although treatment with recombinant human acid α-glucosidase has dramatically improved survival, recombinant human acid α-glucosidase does not reach the brain, and surviving classic infantile Pompe patients develop progressive cognitive deficits and white matter lesions. We investigated the feasibility of measuring non-invasively glycogen build-up and other metabolic alterations in the brain of classic infantile Pompe patients. Four classic infantile patients (8–16 years old) and 4 age-matched healthy controls were scanned on a 7 T MRI scanner. We used T_2_-weighted MRI to assess the presence of white matter lesions as well as ^1^H magnetic resonance spectroscopy and magnetic resonance spectroscopy imaging to obtain the neurochemical profile and its spatial distribution, respectively. All patients had widespread white matter lesions on T_2_-weighted images. Magnetic resonance spectroscopy data from a single volume of interest positioned in the periventricular white matter showed a clear shift in the neurochemical profile, particularly a significant increase in glycogen (result of acid α-glucosidase deficiency) and decrease in *N*-acetyl-aspartate (marker of neuronal damage) in patients. Magnetic resonance spectroscopy imaging results were in line and showed a widespread accumulation of glycogen and a significant lower level of *N*-acetyl-aspartate in patients. Our results illustrate the unique potential of ^1^H magnetic resonance spectroscopy (imaging) to provide a non-invasive readout of the disease pathology in the brain. Further study will assess its potential to monitor disease progression and the correlation with cognitive decline.

## Teaser

Classic infantile Pompe disease is caused by abnormal lysosomal glycogen accumulation in multiple tissues, including the brain. Current treatment does not reach the brain, and patients develop cognitive deficits and brain lesions. Najac *et al*. show the feasibility of using ^1^H magnetic resonance spectroscopy to directly measure glycogen in patients.

## Graphical Abstract

**Graphical Abstract:**
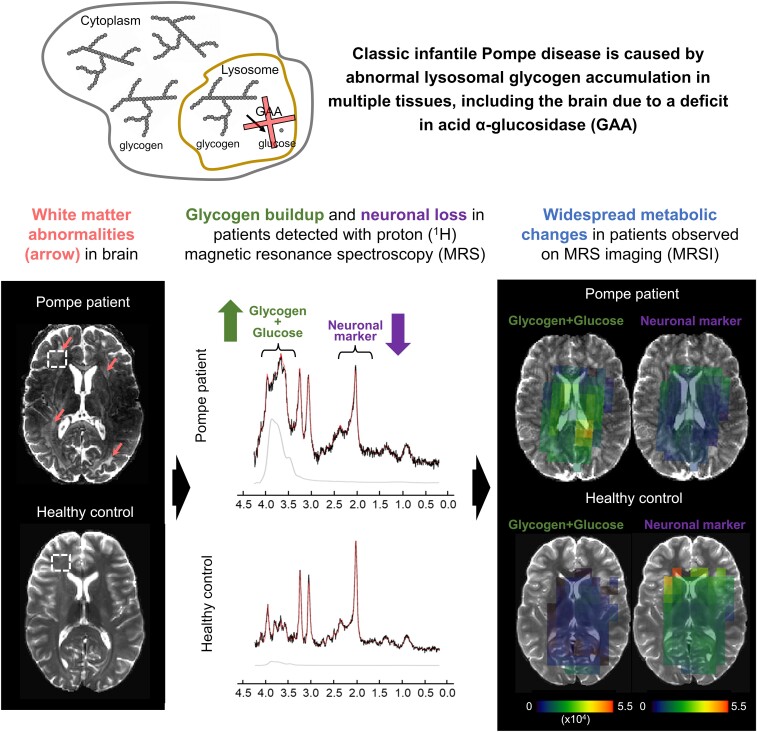

## Introduction

Pompe disease, or glycogen storage disease type II (GSD II), is a rare autosomal recessive disorder caused by a deficiency in the lysosomal enzyme acid α-glucosidase (GAA).^1^ GAA deficiency leads to lysosomal glycogen accumulation in multiple tissues, including the CNS.^1-5^ Pompe disease presents as a clinical spectrum. Patients with classic infantile Pompe disease have no residual enzyme activity and are the most severely affected. They present within the first 6 months of life with a progressive hypertrophic cardiomyopathy, limb weakness and severe hypotonia that prevents them from learning to stand or walk. If untreated, classic infantile patients die before the age of 1 year.^6,7^ Enzyme replacement therapy (ERT) with intravenous recombinant human GAA (rhGAA) reverses cardiomyopathy, enables the achievement of motor milestones and improves survival in classic infantile patients.^8-10^ rhGAA is however limited in its efficacy as it cannot cross the blood–brain barrier (BBB).

Glycogen accumulation occurs beyond the BBB and was described both in various animal models^11-17^ and in autopsies of deceased patients.^4,18^ Studies in animal models of Pompe disease reported pathophysiological alterations, associated with glycogen accumulation, in the cerebrum, cerebellum, brain stem, spiral ganglion, phrenic motoneurons,^12,17^ spinal cord,^13,16^ cochlea^15^ and peripheral nerve and neuromuscular junction.^14^ Reports suggest that therapies targeting muscle alone may be ineffective,^11^ and CNS and muscle targeting treatment studies are currently ongoing in animal models.^19,20^ Post-mortem studies in classic infantile Pompe disease demonstrated that glycogen accumulates in lysosomes within neurons of the brainstem, thalamus, basal ganglia, hippocampus, cerebellum and dentate nucleus as well as in glial cells (mostly astrocytes and microglia) of the entire CNS.^4,18^ Cortical neurons and oligodendrocytes were found to be affected to a lesser extent. As the number of rhGAA-treated classic infantile patients increases over time and the surviving patients become older, clinical consequences potentially related to the glycogen accumulation in the CNS become more and more evident. These vary from hearing problems, fasciobulbar weakness, distal muscle weakness^21-23^ and a decline of processing speed to variations in cognition, ranging from stable and normal to a cognitive decline towards intellectual disability on neuropsychological evaluations (NPEs).^24-28^

MRI has shown white matter (WM) abnormalities in the brain of classic infantile patients, which progressively spread from the frontal and occipital periventricular WM and centrum semiovale to other brain regions.^24^ Additionally, recent findings indicate the occurrence of neuroaxonal damage.^29-31^ Studies using diffusion tensor imaging (DTI) showed a decrease in fractional anisotropy (FA) and an increase in mean diffusivity (MD).^29-31^ In line with these imaging studies, neurofilament light, a protein of the axonal cytoskeleton, deviates from normal in patients after the age of 5 years.^29-31^ Conversely in late-onset patients,^31,32^ who present some residual enzyme activity, no clinically relevant WM abnormalities were observed on conventional anatomical MR images and using DTI. None of those techniques are however able to directly monitor cellular neurochemistry and particularly measure glycogen accumulation.

Proton MR spectroscopy (^1^H MRS) is the most direct method to non-invasively quantify neurometabolite levels.^33^ Of these neurometabolites, *N*-acetyl-aspartate (NAA), typically co-measured with *N*-acetyl-aspartyl-glutamate (NAA + NAAG = tNAA), and glutamate (Glu) are predominantly found in neurons; creatine and phosphocreatine (Cr + PCr = tCr) reside in all neural cells; and soluble choline-containing compounds (tCho) and myo-inositol (Ins) are primarily glial markers.^34^ Changes in metabolite levels have been associated with disease state and are thought to represent different aspects of pathology in many diseases.^35^ For example, lower tNAA levels and Ins levels have been respectively associated with axonal damage and gliosis in adrenoleukodystrophy.^36^ A study conducted at 1.5 T in five classic infantile Pompe patients treated with ERT showed changes in NAA/tCr and NAA/tCho levels that may suggest loss of neuronal viability.^37^ While ^1^H MRS has been used to measure glycogen levels in the liver (200–400 μmol/g),^38,39^ detection of glycogen in the normal brain is all but impossible due to its very low concentration (3–10 μmol/g).^39^ To the best of our knowledge, non-invasive *in vivo* assessment of glycogen in Pompe patients (in the CNS and other organs) has not been reported.

In this study, we used ^1^H MRS to provide a non-invasive readout of glycogen accumulation and other neurochemical alterations in the brain of classic infantile Pompe patients.

## Materials and methods

### Participants

Four classic infantile Pompe patients (age 9–16 years, 2 F/2 M) and 4 age-matched healthy controls (3 F/1 M) participated in this study. Patient diagnosis was confirmed by enzyme activity assays and mutation analysis in the first 6 months after birth. Two patients presented a c.2481 + 102_2646 + 31del538 homozygous mutation and were cross-reactive immunological material (CRIM) positive. One patient was homozygous for c.525delT and CRIM negative. One patient was compound heterozygous for c.2481 + 102_2646 + 31del538 and c.525delT and was CRIM positive. At the time of the MRI, one patient was wheelchair bound (age 13 years), two patients were using the wheelchair outside the house (ages 9 and 16 years) and one patient did not require the use of the wheelchair (age 8 years). Patients were treated with a dose of rhGAA (alglucosidase alfa) of 40 mg/kg/week at the time of the MR scans. All participants gave written informed consent, and the study adhered to the guidelines of the Erasmus University Medical Center and Leiden University Medical Center Institutional Review Boards (The Netherlands).

### NPE

Longitudinal NPEs were conducted at the Erasmus University Medical Center. The Dutch version of the Wechsler Preschool and Primary Scale of Intelligence (WPPSI-III-NL) was used to test children from 2.5 to 6 years, while children with a developmental age of 6–17 years were tested with the Wechsler Intelligence Scale for Children (WISC)-III-NL^40^ or WISC-V-NL.^41^ As described previously,^24^ we could derive total intelligence, verbal intelligence and performance intelligence scores, processing speed, verbal comprehension factor and perceptual organization index. Part of the data presented here have been published as part of other studies.^24,30^ In particular, the NPE data of Patients 3 and 4 have been published up till the age of 10 and 12 years old, respectively.^11^ The total IQ and the processing speed data of all four patients have been compared to the level of neurofilament light.^16^ Due to the small sample size and inherent interindividual variation in NPE results, we chose not to perform statistical analysis. The data are shown as additional information on the cognitive status of the included patients.

### Imaging protocol

**Figure 1:**
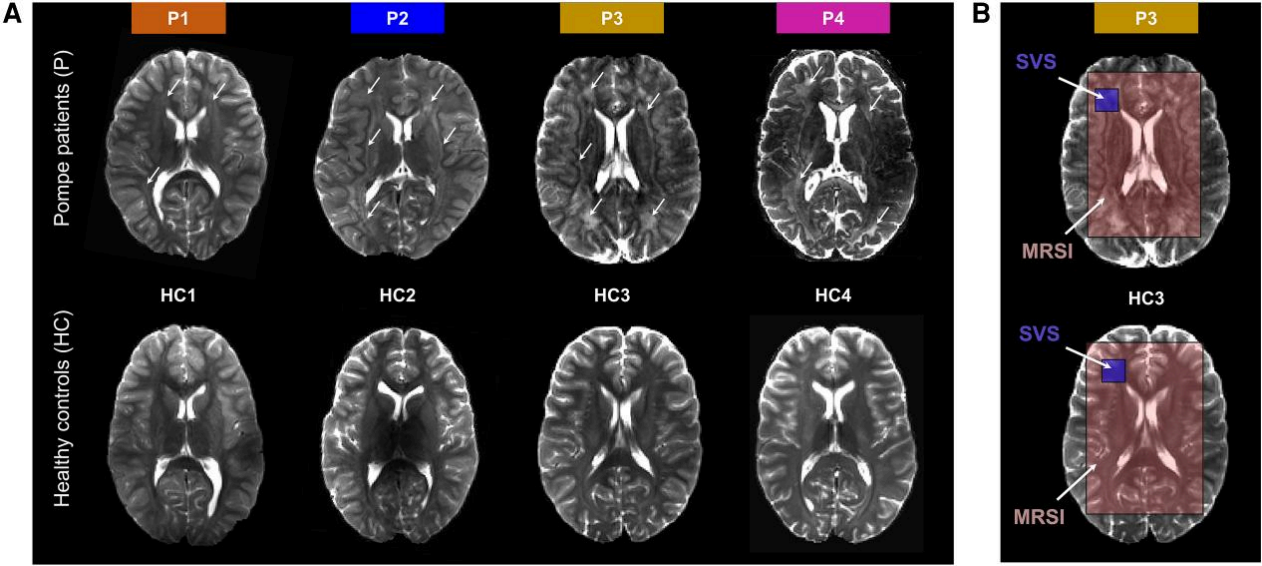
**WM abnormalities in Pompe patients and study planning.** (**A**) T_2_-weighted images of Pompe patients (P1-4, *top*) and age-matched healthy controls (HC1-4, *bottom*), highlighting the WM abnormalities (white *arrows*) in all Pompe patients. (**B**) Example of positioning of the VOI for SVS (blue) and MRSI (red) acquisitions.

All experiments were conducted on a Philips 7 T whole body MRI scanner (Philips Healthcare, The Netherlands) equipped with a quadrature transmit/32-channel receive head coil (Nova Medical, USA) at the Leiden University Medical Center. The scan protocol consisted of a short survey scan (scan time ∼1 min) and a sensitivity encoding reference scan (scan time = 11 s) followed by (Fig. 1): (i) a T_2_-weighted multi-slice multi-echo scan {repetition time [TR]/first echo time [TE]/second TE = 4385/27/100 ms, field of view [anterior–posterior (AP), right–left (RL), front–head (FH)] = 216 × 216 × 80 mm^3^, resolution [AP, RL, FH] 1.3 × 1.3 × 4 mm^3^, scan time ∼6 min}; (ii) 2D Magnetic Resonance Spectroscopic Imaging (MRSI) acquisition (detailed protocol below, scan time ∼11 min); (iii) single-volume spectroscopy (SVS) scans (detailed protocol below, scan time ∼5.2 min); and (iv) 3D T_1_-weighted images [field of view (AP, RL, FH) = 240 × 240 × 150 mm^3^, resolution (AP, RL, FH) 1 × 1 × 1 mm^3^, TR/TE = 5.6/2.6 ms, scan time ∼2 min].

### Single-volume ^1^H MRS acquisitions

SVS data were acquired with a semi-LASER (sLASER) sequence [TR/TE = 6500/34 or 36 ms, number of time domain points (np) = 1024, spectral bandwidth = 3000 Hz, number of sample averages (NSA) = 32]. To minimize in-plane chemical shift displacement errors, frequency offset corrected inversion (FOCI) refocusing pulses were used. Using the T_2_-weighted images, a 18 × 18 × 18 mm^3^ volume of interest (VOI) was positioned in the left frontal periventricular WM region (Fig. 1B). Water suppression was achieved using the variable pulse power and optimization relaxation delays (VAPOR) sequence. Outer volume suppression targeting lipid signal was performed with saturation bands positioned circularly around the VOI.

### 2D ^1^H MRSI acquisitions

A volume selective sLASER sequence (TR/TE = 5000/36 or 38 ms, NSA = 6) was combined with a concentric ring readout trajectory [field of view (AP, RL) = 240 × 240 mm, voxel size: 11.25 × 11.25 mm^2^, slice thickness (FH) = 10 mm, spectral bandwidth = 2003 Hz, train duration = 204.6 ms, np = 400]. Similar to the SVS acquisition, water suppression and outer volume suppression targeting lipids were achieved with VAPOR and saturation bands, respectively. T_2_-weighted images were used to select the region of interest for the 2D MRSI acquisition. In patients, the region of interest included both WM abnormalities and normal-appearing brain tissues, depending on the extent of WM abnormalities (Fig. 1B). Data set from one healthy control was excluded due to subject motion.

### Phantom acquisition

**Figure 2:**
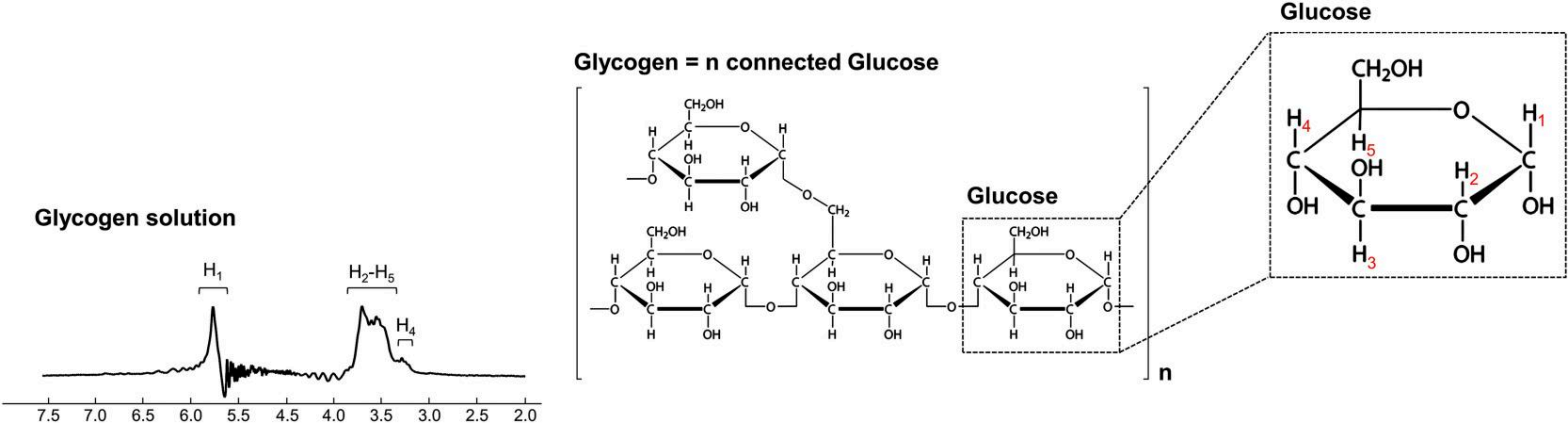
**Glycogen phantom.** A 28 mM glycogen solution phantom was prepared, and single-volume data were collected using the same protocol as for *in vivo* scan. LPSVD Matlab-based routine was used to remove the water residual.

A 28 mM glycogen solution was prepared by dissolving glycogen from rabbit liver (Sigma) in water. Data were acquired using the same sequence parameters used for SVS *in vivo* acquisition, but with a 40 × 16 × 16 mm^3^, NSA = 8352 and np = 2048 (scan time ∼15 h). The spectrum was corrected for frequency drift, and the residual water peak was removed using a Matlab-based linear prediction singular value decomposition (LPSVD) routine (Fig. 2).

### Data processing

Anatomical images were assessed for WM abnormalities and scored by a neuroradiologist (Supplementary Table 1). The reconstruction and processing of SVS and 2D MRSI data were performed using custom-written Matlab (The MathWorks, Inc., Natick, MA) routines as previously described.^42^ Spectra were fitted using LCModel^43^ with respective basis sets. To fit the *in vivo* glycogen signal, the phantom spectrum was added to the LCModel basis sets. As glucose and glycogen fits were largely correlated in classic infantile patients due to the large overlaps in their proton resonances, we here report the sum of glycogen and glucose (Glyc + Glc).

### Statistical analysis

Due to our small sample size, statistical significance was tested using a one-tailed Mann–Whitney U-test (*P < 0.05, GraphPad Software, USA). For SVS data, we compared metabolite ratios between patients and healthy controls. For MRSI data, we averaged metabolite ratios across all voxels for each individual and compared the averaged values between healthy controls and patients. We also performed a Spearman correlation between Glyc + Glc/tCr and tNAA/tCr across all voxels in all patients and fitted the data with linear regression.

## Results

### Patients show WM abnormalities and cognitive decline

**Figure 3:**
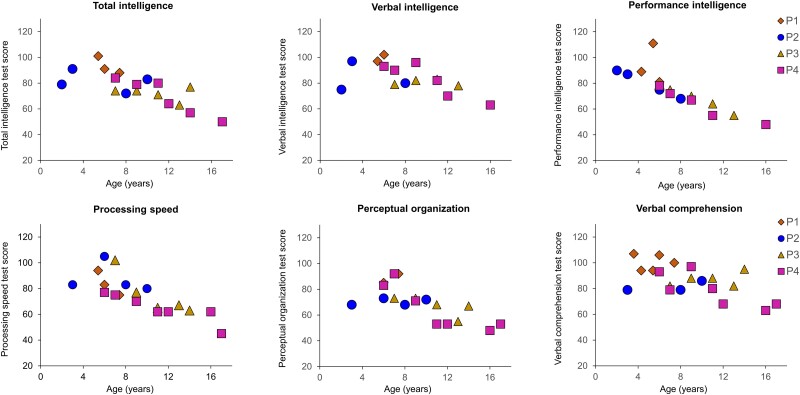
**Neuropsychological test scores over time for all four patients.** Patient 1 (P1), orange diamond. Patient 2 (P2), blue circle. Patient 3 (P3), yellow triangle. Patient 4 (P4), pink square.

As illustrated in Fig. 1A and reported in Supplementary Table 1, all Pompe patients presented WM abnormalities on conventional T_2_-weighted images at time of the MRI. All patients showed WM abnormalities in the frontal, parietal and occipital subcortical areas as well as in the capsula externa. Some regions (corticospinal tracts, midbrain and cerebellum) were not covered by the T_2_w images in all patients and could not be evaluated. As summarized in Fig. 3, Patients 1 and 4 showed a decline of 1 SD or more in processing speed and Patients 2 and 4 showed a decline in total intelligence and performance intelligence scores over time.

### Phantom data provided a basis to quantify glycogen *in vivo*

As illustrated in Fig. 2, a good-quality spectrum of glycogen solution was obtained using our MRS sequence parameters. A large resonance between 3.2 and 3.8 ppm, corresponding to the H_2_-H_5_ and H_4_ protons, and a narrower resonance at 5.8 ppm, corresponding to the H_1_ proton group were detected. The spectrum was added to the LCModel basis set to allow quantification of glycogen *in vivo*.

### Altered glycogen and total NAA levels in WM abnormalities

**Figure 4:**
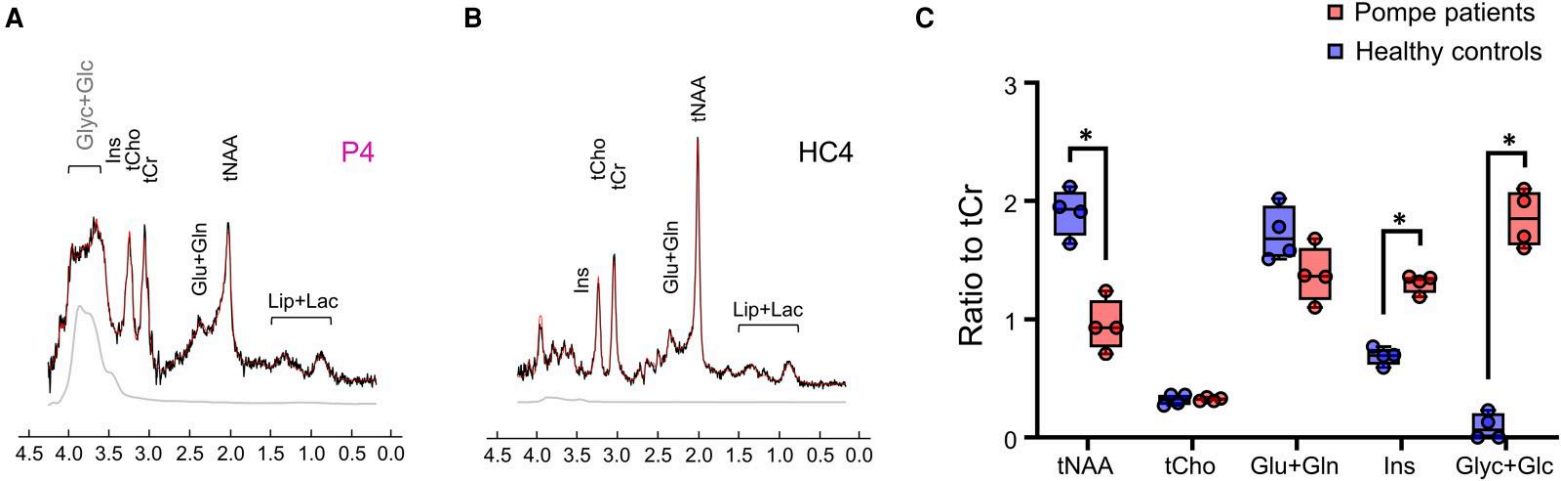
**Neurochemical changes in Pompe patients’ WM abnormalities.** Example of SVS data acquired in (**A**) P4 (Patient 4) and (**B**) HC4 (Healthy Control 4) illustrating the clear change in neurochemical profile, particularly in Glyc + Glc (3.2–3.8 ppm region) and tNAA (2 ppm). (**C**) Quantification showing significant alteration in the neurochemical profile in Pompe disease. Statistical significance (represented with *) was evaluated using a Mann–Whitney test and with *P < 0.05. For display, the amplitude of the spectra shown in **A** and **B** was normalized to the level of tCho of each subject. Glyc + Glc, glycogen + glucose; Ins, myo-inositol; tCho, choline-containing compounds; tCr, total creatine = creatine (Cr) + phosphocreatine (PCr); Glu + Gln, glutamate + glutamine; tNAA, total NAA = NAA + NAAG; Lip + Lac, lipid + lactate.

Single-volume ^1^H MRS data showed a clear shift in the neurochemical profile in Pompe patients in a VOI with WM abnormalities (Fig. 4A and B, Supplementary Figs 1 and 2). While the H_1_ glycogen peak (at 5.8 ppm) was not detected *in vivo*, potentially due to broadening of the H_1_ peak *in vivo* and artefacts due to water signal suppression, the signal at 3.2–3.8 ppm was clearly visible in all patients (Supplementary Fig. 2). When fitting the acquired data (Supplementary Fig. 3, Fig. 4C), we measured significantly lower tNAA/tCr ratio (*P* = 0.014) and significantly higher Ins/tCr ratio (*P* = 0.014) and (Glyc + Glc)/tCr ratio (*P* = 0.014) in patients than in healthy controls. Full report of Cramér–Rao lower bound (CRLB) values and concentrations for all metabolites can be found in Supplementary Table 2 in the Supplementary material.

### 2D^1^H MRSI shows widespread differences in tNAA and Glyc + Glc levels between patients and healthy controls

**Figure 5:**
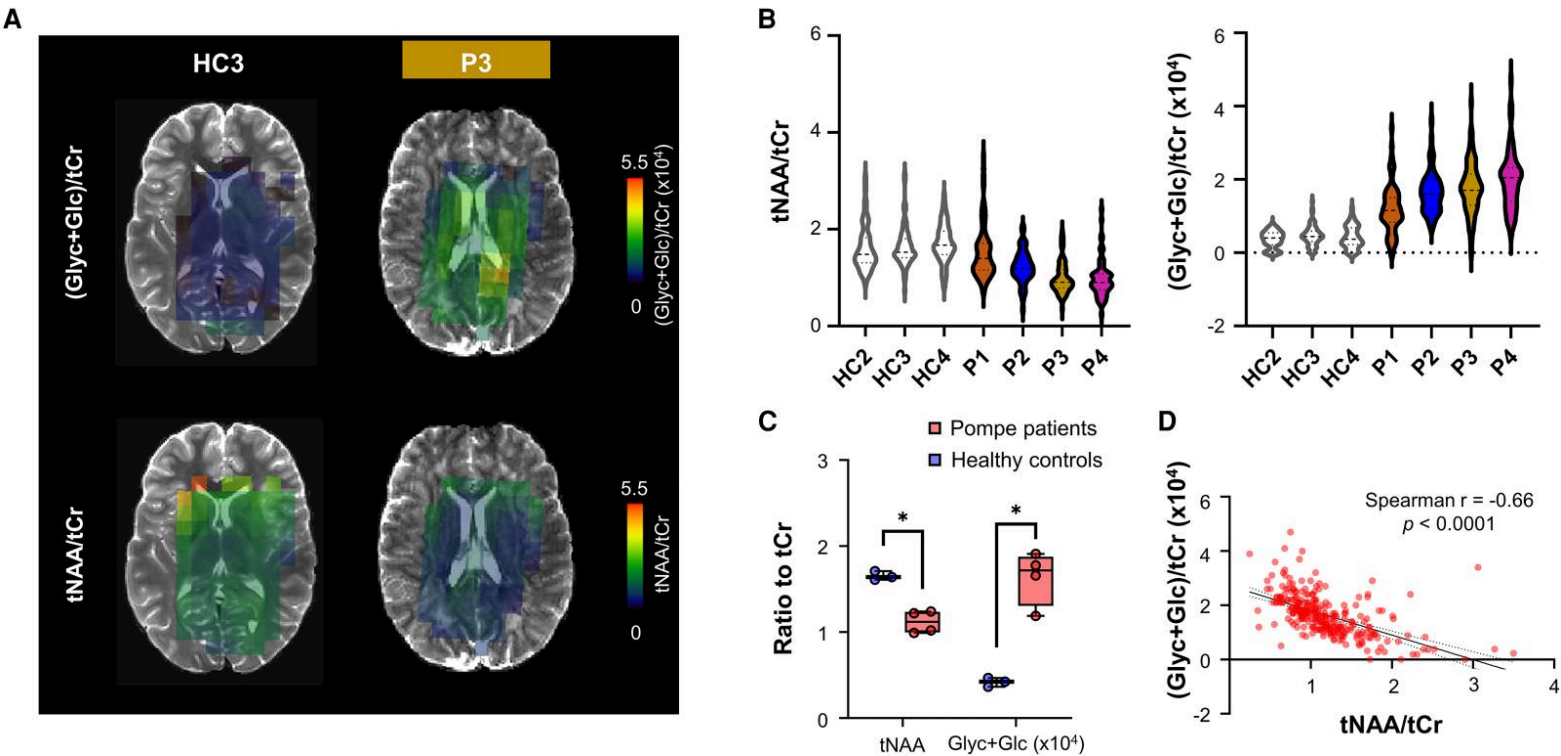
**(Glyc + Glc)/tCr and tNAA/tCr maps in Pompe patients.** (**A**) Example of (Glyc + Glc)/tCr (*top*) and tNAA/tCr (*bottom*) maps obtained following LCModel fitting in one healthy control (HC3, *left*) and one Pompe patient (P3, *right*). A threshold was applied to remove spectra with low SNR or large lipid contamination. (**B**) Violin plots showing the distribution of (Glyc + Glc)/tCr (*right*) and tNAA/tCr (*left*) across all voxels for all individuals. The total of voxels included per subject was: 54/76/53/76/54/77/70 for HC2/HC3/HC4/P1/P2/P3/P4, respectively. (**C**) Plots showing the means of (Glyc + Glc)/tCr (*right*) and tNAA/tCr (*left*) across all voxels in healthy controls (blue) versus Pompe patients (red). Data set from one healthy control was excluded due to subject motion. (**D**) Correlation between (Glyc + Glc)/tCr and tNAA/tCr levels in all voxels in all Pompe patients is shown. The solid line shows the linear regression, and the dashed lines represent the 95% confidence intervals (*R*^2^ = 0.32, *P* < 0.0001). Statistical significance (represented with *) was evaluated using a Mann–Whitney test and with *P < 0.05. HC2-4 refers to Healthy Control 2-4, and P1-4 refers to Patient 1-4.

As illustrated in Fig. 5A and Supplementary Fig. 3, the neurochemical profile in Pompe patients is affected throughout the region covered by the 2D MRSI acquisition. Figure 5B shows the distribution of tNAA/tCr and (Glyc + Glc)/tCr across all voxels for all individuals, illustrating that the median values of tNAA/tCr are lower and those of (Glyc + Glc)/tCr are higher, respectively, in all patients than controls. As shown in Fig. 5C, a significantly higher (Glyc + Glc)/tCr was found in patients (*P* = 0.0286) as well as a significantly lower tNAA/tCr (*P* = 0.0286). Figure 5D shows the relation between tNAA/tCr and (Glyc + Glc)/tCr levels in Pompe patients (*r* = −0.66, *P* < 0.0001). Full report of CRLB values and concentrations for all metabolites can be found in Supplementary Table 3 in the Supplementary material.

## Discussion

We demonstrated the feasibility of using ^1^H MRS at ultrahigh field (7 T) for detecting glycogen deposition and assessing alterations in the neurochemical profile in the brain of classic infantile Pompe patients. The most salient finding is the detection of an elevated signal in the 3.2–3.8 ppm chemical shift range, which we attribute to glycogen, in the brain of patients, and concomitantly significantly lower tNAA levels (Figs 4 and 5). This finding was consistent throughout all four patients.

Classic infantile Pompe patients are the most severely affected patients in the Pompe disease spectrum and have virtually no residual GAA activity.^1-7^ ERT is currently the only treatment for Pompe disease; however, it does not cross the BBB and does not treat the CNS pathology. As classic infantile patients now survive thanks to ERT, progressive WM abnormalities and cognitive problems become apparent. Innovative treatment strategies that are currently under development therefore aim to target both muscles and the brain.^24-26^ The non-invasive approach to detect glycogen accumulation in infantile Pompe disease patients that we show here illustrates the unique potential of ^1^H MRS(I) to offer a possible direct, non-invasive readout of disease progression and a possible tool to measure the response to treatment in the CNS.

Detection of glycogen using ^1^H MRS has previously been demonstrated in phantom^44^ and in the human liver.^38,44^ In the healthy human brain, the low levels of glycogen and its short spin–spin relaxation time (T_2_) make glycogen detection with MRS essentially impossible.^45^ In our MRS measurements on a glycogen phantom, we were able to detect the signal from the H_3_-H_5_ resonances (between 3.2 and 3.8 ppm) (Fig. 2). We used data acquired in a glycogen phantom to fit *in vivo* data (Fig. 4, Supplementary Fig. 3). This allowed us to account for the increase in signal in the 3.2–3.8 ppm chemical shift range in all four patients. It is difficult to fully disentangle the contributions of glycogen and glucose to the signal and attribute the increase in signal to glycogen alone. Nonetheless, the accumulation of glycogen we observed is in line with previous post-mortem studies showing deposition of glycogen in various cell types and brain regions in classic infantile patients.^4,5^

Lower levels of tNAA, a surrogate measure of mitochondrial and neuronal viability, suggest widespread neuronal dysfunction, which is in line with a previous study.^37^ It is also in agreement with neuroaxonal damages detected with DTI and changes in neurofilament light levels.^29-31^ We also observed a higher level of Ins, a glial marker, in the patient population. However, the partial overlap of Ins, Glyc and Glc resonances on the ^1^H MR spectrum might bias the quantification of Ins towards an overestimation, a marker of neuroinflammation.

Similar to previous findings,^24,25,29,46^ all four patients showed a decrease of procession speed, as shown by the NPEs (Fig. 3), as well as relatively widespread WM hyperintensities on T_2_-weighted images^24,25,29,46^ (Fig. 1).

A limitation of our study is the relatively small size of our group, associated with the rarity of Pompe disease. All four patients, however, showed similar metabolic alterations. Our measurements were performed using ultrahigh field MRI scanner (7 T), resulting in a higher signal-to-noise ratio compared to similar acquisitions on a clinical scanner operating at 3 or 1.5 T. The Glyc + Glc signal we detected in all four patients was very high, with very low uncertainty per subject. This suggests that glycogen quantification with MRS is possible at clinical field strengths (3 T), and further studies are needed to assess the accuracy of glycogen quantification at fields lower than 7 T. If so, this could allow including MRS(I), together with other biomarkers (e.g. FA/MD obtained with DTI and measurement of neurofilament light measured in serum), in follow-up programmes to capture the CNS involvement in larger, international patient cohorts. This could help to establish a benchmark to evaluate response to next-generation therapies that include the brain as a target.

The first radiological signs of brain involvement seen so far in patients with classic infantile Pompe disease are WM abnormalities on structural MRIs. Recent insights in WM disease showed that impaired conduction can lead to a decline of processing speed, slowed cognition and impaired visuospatial functioning, which are also reported in patients with classic infantile Pompe disease.^24,26^ Nevertheless, abnormalities in the basal ganglia—subcortical grey matter (GM)—were also reported. GM involvement typically leads to deficits in higher cortical functions such as language that are not seen in classic infantile patients. While GM neuropathology may add to cognitive problems, more insight is needed into the extent and timing of WM and GM involvement in classic infantile Pompe disease and their effect on cognitive functioning. In this study, we chose to focus on a single-volume measurement in a region affected with WM abnormalities and on 2D MRSI measurement in areas with substantial amount of WM abnormalities in all four patients. In a future study, we will focus on areas with less or no WM abnormalities to evaluate if glycogen accumulation precedes WM lesions.

All patients included in our study were older than 9 years and showed WM abnormalities on anatomical MR images. One patient showed signs of atrophy. Including younger patients in future studies will allow evaluating the potential relation in time between the development of CNS abnormalities and glycogen accumulation at a younger age. Performing MRI scans on young children can be challenging; however, reports show that the use of a mock scanner to explain and train children (with an age between 4 and 7 years) can greatly reduce anxiety and enable cooperation.^47^

A longitudinal follow-up study we plan to perform will inform on the correlation between glycogen deposition, measured by MRS, and the extent of cognitive problems and WM abnormalities. Although no clinically relevant WM abnormalities were observed so far in the brain of late-onset patients, a recently published case study in a patient with late-onset Pompe disease did report atrophy of the motoneurons in the medulla and rostral spinal cord as well as ballooning of the lumbar and sacral motoneurons as seen in patients with classic infantile Pompe disease.^48^ This prompts us to investigate if glycogen could be directly (or not) detected with MRS(I) in late-onset patients.

In conclusion, by offering a direct, non-invasive and *in vivo* outcome measure of brain involvement in Pompe disease, the use of MRS(I) to monitor glycogen deposition could help to set a benchmark and to serve as an outcome marker in future clinical trials with next-generation therapies.

## Supplementary Material

fcae303_Supplementary_Data

## Data Availability

Anonymized data are available from the corresponding author on request by a qualified academic investigator. Data transfer will conform with EU legislation on the general data protection regulation and decisions by the Ethical Review Boards of the Erasmus University Medical Center and Leiden University Medical Center.
