# Supplementary material for: Brain glycogen build-up measured by magnetic resonance spectroscopy in classic infantile Pompe disease
Source: Brain Commun. 2024 Sep 12;6(5):fcae303. doi: 10.1093/braincomms/fcae303 (PMC11416038; doi:10.1093/braincomms/fcae303)
Supplement: fcae303_Supplementary_Data [file fcae303_supplementary_data.docx]

Brain glycogen buildup measured by magnetic resonance spectroscopy in classic infantile Pompe disease

Chloé Najac,^1^ Nadine A.M.E. van der Beek,^2^ Vincent O. Boer,^3^ Pieter A. van Doorn,^2^ Ans T. van der Ploeg,^4^ Itamar Ronen,^5^ Hermien E. Kan^1,6^ and Johanna M.P. van den Hout^4^

**- Supplementary Material -**

**Supplementary Table 1** Results of the standardized scoring of MRI abnormalities in patients with classic-infantile Pompe disease. 0 = normal, 1 = abnormal, NA = not available, WMA = white matter abnormalities, PLIC = posterior limb of internal capsule, ALIC = anterior limb of internal capsule.

| **Regions** | **P1** | **P2** | **P3** | **P4** |
| --- | --- | --- | --- | --- |
| WHA Frontal Subcortical | 1 | 1 | 1 | 1 |
| WMA Frontal U-fibres | 0 | 0 | 0 | 1 |
| WMA Parietal Subcortical | 1 | 1 | 1 | 1 |
| WMA Parietal U-fibres | 0 | 0 | 0 | 1 |
| WMA Temporal Subcortical | NA | 1 | NA | 1 |
| WMA Temporal U-fibres | NA | 0 | NA | 1 |
| WMA Occipital Subcortical | 1 | 1 | 1 | 1 |
| WMA Occipital U-fibres | 0 | 0 | 0 | 1 |
| Corpus Callosum | 1 | 0 | 0 | 1 |
| Capsula Externa | 1 | 1 | 1 | 1 |
| PLIC | 0 | 1 | 1 | 1 |
| ALIC | 0 | 0 | NA | 1 |
| Corticospinal tracts | 0 | 1 | NA | 1 |
| Midbrain/Pons/Medulla/Thalamus | 0 | 0 | NA | NA |
| Basal Ganglia | 0 | 0 | 1 | 1 |
| Cerebellum | NA | NA | NA | 1 |

**Supplementary Table 2** SNR, FWHM, concentration normalized to tCr and CRLB values (mean±s.d.) from SVS measurement are reported.

|  | Classic-infantile Pompe patients | | Age-matched healthy controls | |
| --- | --- | --- | --- | --- |
| SNR  FWHM | 19±5  19.5±6.4 Hz | | 27±6  16.8±4.3 Hz | |
|  | **/Cr** | **CRLB (%)** | **/Cr** | **CRLB (%)** |
| tNAA | 0.95±0.22 | 2.00±0.00 | 1.91±0.20 | 1.25±0.50 |
| tCho | 0.32±0.02 | 2.25±0.50 | 0.32±0.05 | 2.50±0.58 |
| Glu | 0.69±0.20 | 6.75±2.06 | 1.19±0.13 | 4.50±1.00 |
| Gln | 0.68±0.03 | 8.25±0.96 | 0.53±0.12 | 13.25±2.75 |
| Glx | 1.38±0.24 | 3.25±0.96 | 1.72±0.23 | 4.50±0.58 |
| Ins | 1.30±0.08 | 3.00±1.41 | 0.69±0.07 | 4.00±0.50 |
| Glyc+Glc  (x10^4^) | 1.85±0.24 | 3.50±0.71 | 0.09±0.03 | 1116.52±481.61 |

**Supplementary Table 3** SNR, FWHM, concentration normalized to tCr and CRLB values (mean±s.d.) from MRSI measurement (across all voxels) are reported.

|  | Classic-infantile Pompe patients | | Age-matched healthy controls | |
| --- | --- | --- | --- | --- |
| SNR  FWHM | 16±5  21.2±6.2 Hz | | 19±6  19.8±5.3 Hz | |
|  | **/Cr** | **CRLB (%)** | **/Cr** | **CRLB (%)** |
| tNAA | 1.18±0.48 | 3.74±2.03 | 1.65±0.42 | 2.83±1.18 |
| tCho | 0.21±0.06 | 16.22±84.30 | 0.21±0.06 | 8.34±5.99 |
| Glu | 0.96±0.36 | 17.92±87.30 | 1.10±0.26 | 8.23±6.78 |
| Gln | 0.63±0.28 | 41.95±146.03 | 0.37±0.22 | 79.37±183.00 |
| Glx | 1.59±0.50 | 8.17±4.66 | 1.47±0.37 | 8.71±4.21 |
| Ins | 1.19±0.29 | 4.31±2.11 | 0.63±0.14 | 8.32±20.25 |
| Glyc+Glc  (x10^4^) | 1.65±0.76 | 9.48±11.82 | 0.42±0.0.27 | 53.17±139.37 |

**Supplementary Figure 1** T_2_-weighted images for **(a)** Pompe patient (P3) and **(b)** age-matched healthy control (HC3) with overlaid VOI. Single-volume MRS data acquired in **(c)** all Pompe patients and **(d)** age-matched healthy controls showed a significant alteration in the neurochemical profile in Pompe disease.


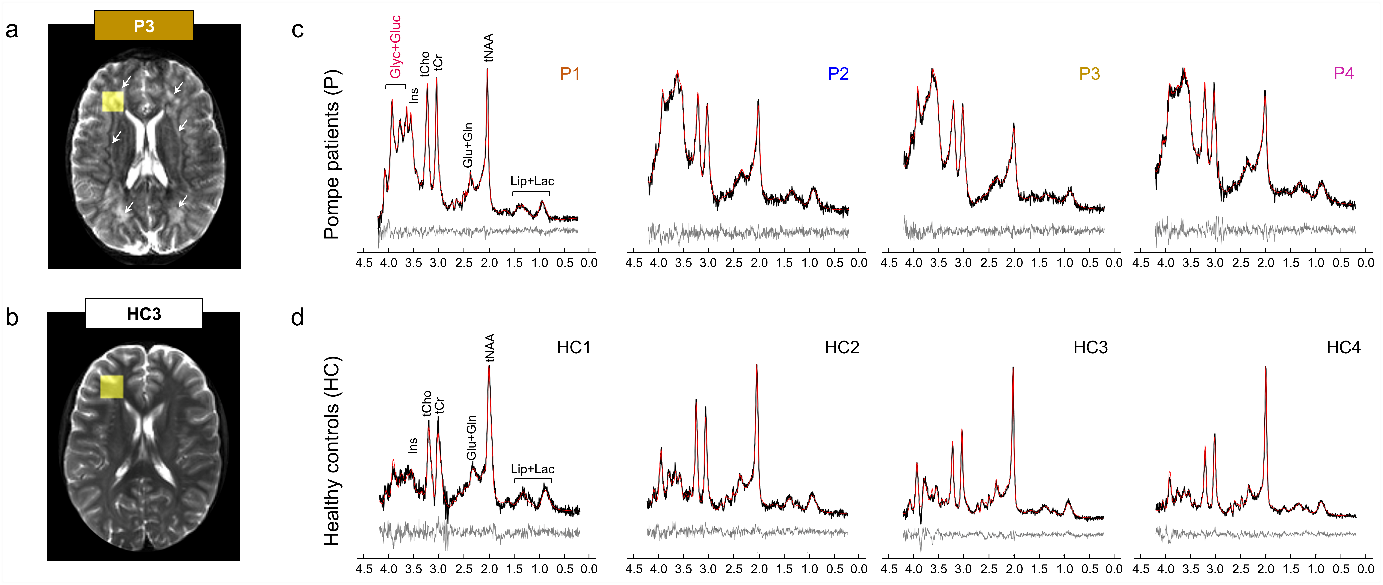


**Supplementary Figure 2** T_2_-weighted images for **(a)** Pompe patient (P2) and **(b)** age-matched healthy control (HC2) with overlaid VOI. Spectral fit of single-volume MRS spectra from **(c)** Pompe patient (P2) and **(d)** age-matched healthy control (HC2).

**
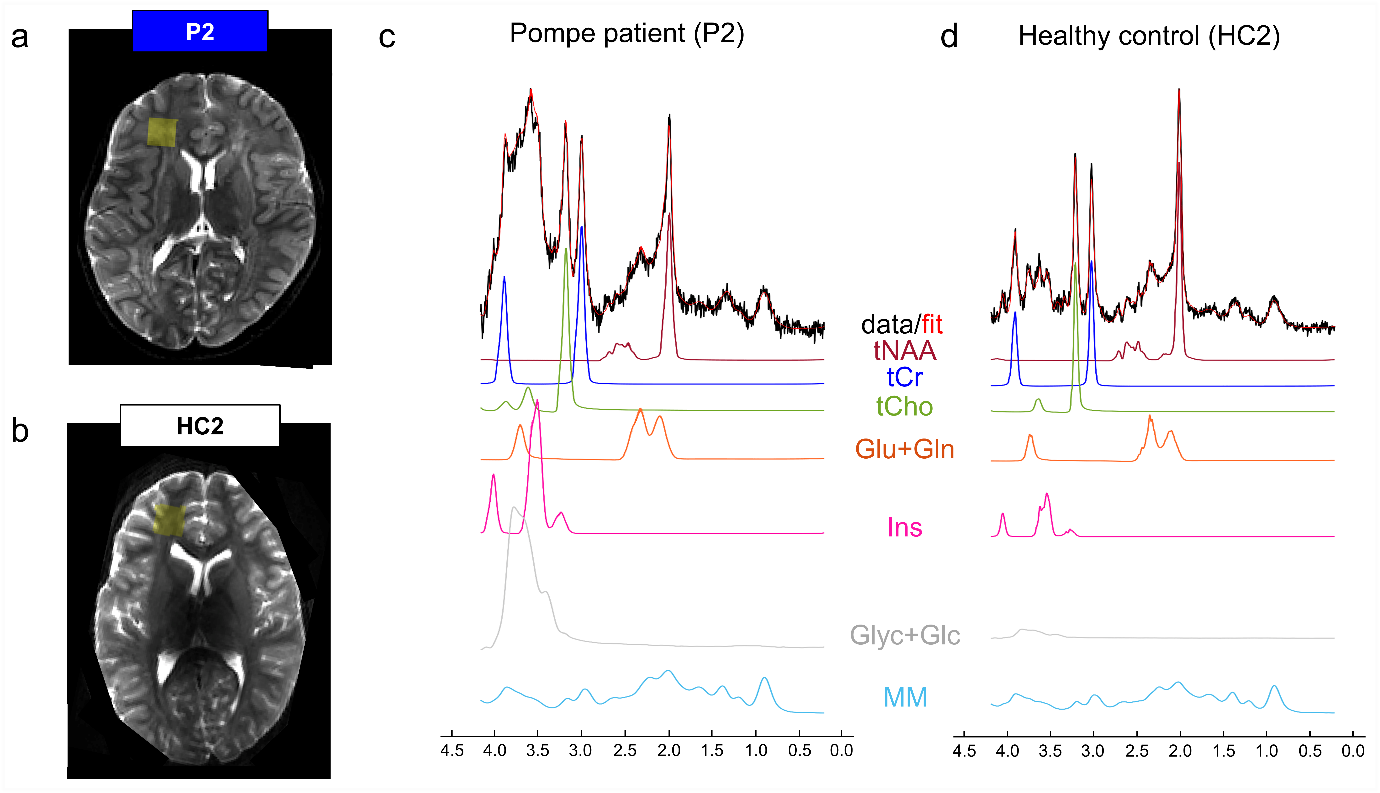
**


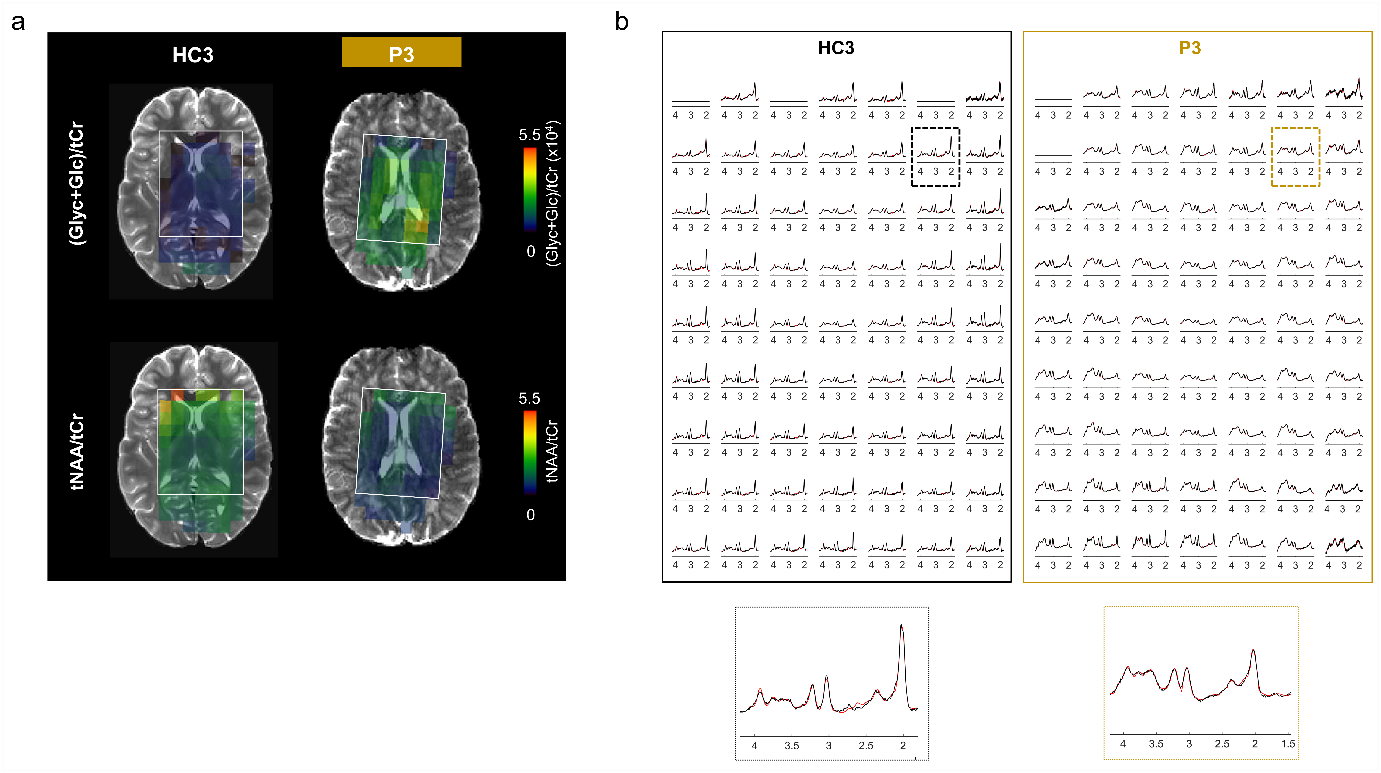
**Supplementary Figure 3** **(a)** Example of (Glyc+Glc)/tCr (top) and tNAA/tCr (bottom) maps obtained following LCModel fitting in one healthy control (left, HC3) and one Pompe patient (right, P3). A threshold was applied to remove spectra with low SNR or large lipid contamination. **(b)** Example of spectra data obtained within white box shown in panel A for both individuals.
